# *In vitro* and *in vivo* effects of suloctidil on growth and biofilm formation of the opportunistic fungus *Candida albicans*

**DOI:** 10.18632/oncotarget.19542

**Published:** 2017-07-25

**Authors:** Beini Zeng, Jiachen Li, Yajie Wang, Pengxiang Chen, Xiaohong Wang, Jianfeng Cui, Lidong Liu, Xiaoyan Hu, Qian Cao, Ying Xiao, Junlu Dong, Yundong Sun, Yabin Zhou

**Affiliations:** ^1^ Department of Pathogenic Biology and Key Laboratory for Experimental Teratology of Chinese Ministry of Education, School of Basic Medical Sciences, Shandong University, Jinan, Shandong 250012, People’s Republic of China; ^2^ Department of Radiation Oncology, Qilu Hospital of Shandong University, Jinan, Shandong 250012, People's Republic of China; ^3^ Department of Urology, Qilu Hospital of Shandong University, Jinan, Shandong 250012, People's Republic of China; ^4^ Department of Obstetrics and Gynecology, Qilu Hospital of Shandong University, Jinan, Shandong 250012, People's Republic of China; ^5^ Department of Neurobiology, School of Basic Medical Sciences, Shandong University, Jinan, Shandong 250012, People’s Republic of China

**Keywords:** C. albicans, biofilm, suloctidil, virulence, vaginal candidiasis

## Abstract

As the most frequent fungal pathogen in humans, *Candida albicans* can develop serious drug resistance because its biofilms are resistant to most antifungal agents; this leads to an urgent need to develop novel antifungals. Here, we evaluated the efficacy of an antithrombotic drug, suloctidil, against *C. albicans* biofilms *in vitro* and *in vivo*. We found that suloctidil is effective to inhibit *C. albicans* biofilm, with a minimum inhibitory concentration (MIC_80_) of 4 μg/mL, a biofilm inhibiting concentration (BIC_80_) of 16 μg/mL and a biofilm eradicating concentration (BEC_80_) of 64 μg/mL. Furthermore, the concentration-dependent characteristics of suloctidil were shown by its time-kill curves. Scanning electron microscopy images clearly revealed the morphological effects of suloctidil on biofilm. Yeast-to-hyphal form switching is a key virulence factor of *C. albicans*; therefore, we performed hyphal growth tests and observed that suloctidil inhibited yeast-to-hyphal form switching. This result was consistent with the down-regulation of hypha-specific gene (*HWP1, ALS3*, and *ECE1*) expression levels after suloctidil treatment. *In vivo*, 256 μg/mL of suloctidil significantly reduced fungal counts (*P*<0.01) compared to that in groups without treatment; the treatment group induced a slight histological reaction, especially when the treatment lasted for 5 days (*P*<0.01). Taken together, our data suggest that suloctidil is a potential antifungal agent.

## INTRODUCTION

*Candida. albicans* is an opportunistic pathogen found in approximately 30%–70% of individuals; this pathogen asymptomatically colonizes the skin, mouth, vaginal mucosa, and gastrointestinal tract and causes candidiasis [1–3]. According to the Centers for Disease Control, oral candidiasis develops in approximately 7% of infants, 31% of AIDS patients, and 20% of cancer patients receiving chemotherapy [4]. Vulvovaginal candidiasis is more common and is estimated to occur more than once in 75% of healthy women [5]. Invasive candidiasis has a high mortality of 40%–60% [6] because *C. albicans* adapts to various environmental conditions by developing biofilms, which encase themselves in a self-released slimy polysaccharide and protein layer that enables *C. albicans* to adhere to surfaces [6–8]. The formation of *C. albicans* biofilms includes adhesion, cell growth, initial colonization, and maturation phases [8]. Biofilm formation is an important virulence factor of *C. albicans* and occurs in host tissues, prostheses, and in-dwelling medical devices, such as urinary and vascular catheters, (dental) implants, and heart valves [6, 9–11]. The biofilm significantly increases the resistance of *C. albicans* to traditional antifungal drugs, biofilm-associated *C. albicans* is 4000 times more resistant to fluconazole than *C. albicans* in its planktonic form [6, 12]. Hence, alternatives to conventional antifungal agents are urgently needed to damage *C. albicans* biofilms.

Suloctidil (1-(4-isopropylthiophenyl)-2-n-octylaminopropanol) is a vascular antispasmodic and antithrombotic drug [13] with *in vitro* antifungal properties against *C. albicans* [14]. However, the activity of suloctidil against *C. albicans* biofilms has not been studied. The present study aims to explore the inhibitory effect of suloctidil on the formation of *C. albicans* biofilm and on preformed biofilm to evaluate its potential therapeutic application in biofilm-associated candidiasis.

## RESULTS

### Antifungal activity of suloctidil

MIC_80_ is defined as the lowest concentration of suloctidil that inhibited 80% of cell growth compared to the control (without suloctidil). The MIC_80_ of YEM30 and LC30 was 4 μg/mL (Table 1). A higher concentration completely inhibited their growth.

**Table 1:** Antifungal activity of suloctidil against planktonic cells and biofilms of *C. albicans* strains YEM30 and LC3

Drug concentration and strain	MIC_80_(μg/mL)	BIC_80_(μg/mL)	BEC_80_ (μg/mL)
YEM30	4	16	64
LC3	4	16	64

Antifungal activity of suloctidil against planktonic cells and biofilms of *C. albicans* strains YEM30 and LC3.

### Inhibition of suloctidil on biofilm formation and preformed biofilm

**Figure 1:**
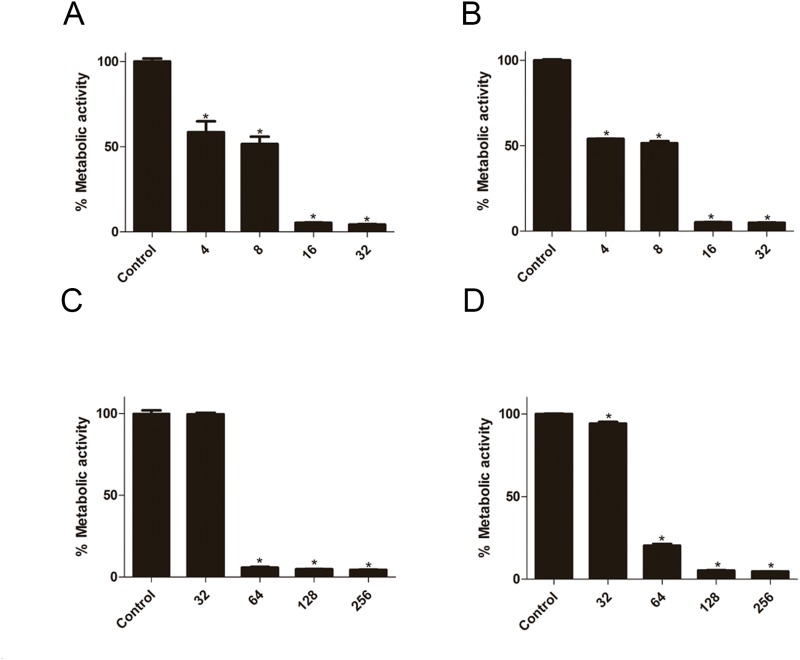
Effect of suloctidil on *C. albicans* biofilms Effect of various suloctidil concentrations on biofilm formation of *C. albicans* YEM30 **(A)** and LC3 **(B)**. Effect of suloctidil on preformed biofilms of YEM30 **(C)** and LC3 **(D)**. The untreated group was set as the control. *P < 0.05; **P < 0.01.

The effect of suloctidil on biofilm formation was evaluated by BIC_80_ (biofilm inhibiting concentration), which is defined as the lowest concentration of suloctidil that inhibited 80% of the metabolic activity of the biofilm formation compared with the control. We found that the BIC_80_ of YEM30 and LC30 was 16 μg/mL (Table 1, Figure 1). The effect of suloctidil on the preformed biofilm was evaluated by BEC_80_ (biofilm-eradicating concentration), which is defined as the lowest concentration of suloctidil that eradicated 80% of the biofilm compared to the control. The BEC_80_ for YEM30 and LC30 was 64μg/mL (Table 1, Figure 1).

### Time-kill curve

**Figure 2:**
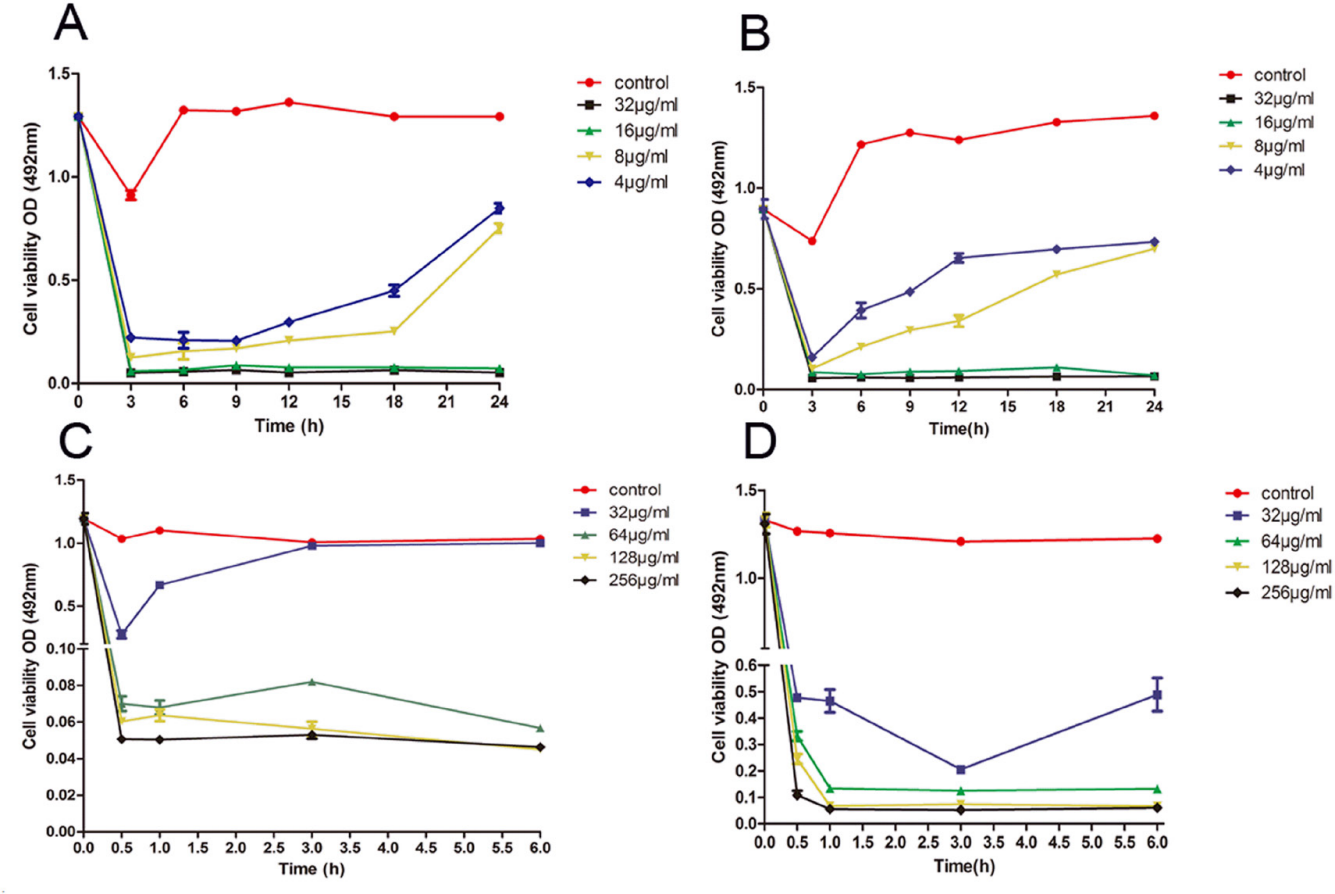
Time–kill curve of suloctidil against *C. albicans* biofilms Representative time-kill curves of 4 μg/ml, 8 μg/ml, 16 μg/ml, and 32 μg/ml suloctidil on biofilm formation of *C. albicans* YEM30 **(A)** and LC3 **(B)**. Representative time-kill curves of 32 μg/mL, 64 μg/mL, 128 μg/mL, and 256 μg/mL suloctidil on preformed biofilms of YEM30 **(C)** and LC3 **(D)**. The untreated group was used as the control.

To further study the kinetics of the anti-biofilm activity of the drugs, we performed the time-kill curve assay, which provides growth kinetic information over time and a more detailed picture of the effects of drugs on cell viability. Thus, the time-kill curve assay significantly enhances our understanding of the dynamic relationships between antifungals and *C. albicans* [24, 26]. Suloctidil exhibited dose-dependent activity against the two strains (Figure 2). In YEM30, the inhibitory activity of suloctidil against biofilm formation weakened the exposure over 6 h for sub-BIC (4 μg/mL) and 12 h for sub-BIC (8 μg/mL) (Figure 2A), respectively, and 6 h for both sub-BIC (4 μg/mL and 8 μg/mL) in LC3 (Figure 2B). Suloctidil (16 and 32 μg/mL) completely inhibited biofilm formation, and the fungicidal endpoint for YEM30 and LC3 was achieved after 3 h at BIC (16 μg/mL) and 2 × BIC (32 μg/mL) of suloctidil (Figure 2A and 2B).

**Figure 3:**
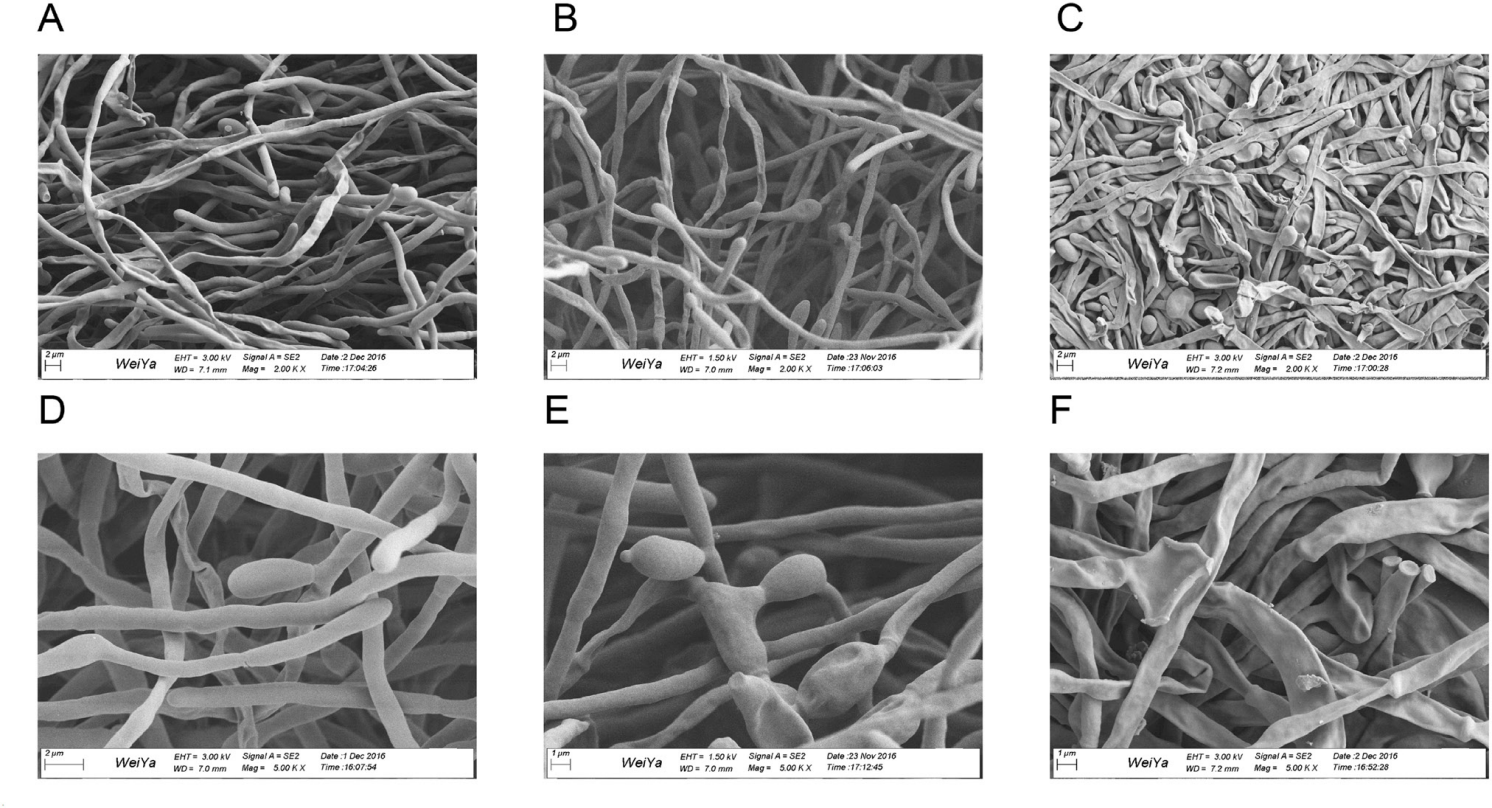
Scanning electron microscopy images of *C. albicans* biofilms Images of preformed biofilms of *C. albicans* YEM30 treated with suloctidil (64 μg/mL) for 1 h **(B, E)** and 24 h **(C, F)**. The untreated group was used as the control **(A, D)**.

In addition, suloctidil (64, 128, and 256 μg/mL) rapidly exerted its activity against the mature-phase biofilm in both strains. Less than 0.5 h was needed to arrive at the fungicidal endpoint (Figure 2C and 2D), and the inhibitory activity of suloctidil against the preformed biofilm weakened the exposure at 0.5 h for sub-BEC (32 μg/mL) in YEM30 (Figure 2C) and 3 h for sub-BEC (32 μg/mL) in LC3 (Figure 2D).

### Scanning electron microscopy

SEM images are shown in Figure 3. Compared with the control group (Figure 3A and 3D), the biofilm exposed to 64 μg/mL of suloctidil for 1 h (Figure 3B and 3E) showed slightly shrunken budding cells. After 24 h, the treated biofilm (Figure 3C and 3F) showed obvious cellular damage: cells were completely shriveled, and budding cells had fallen off the hyphae.

### Hyphal growth test

**Figure 4:**
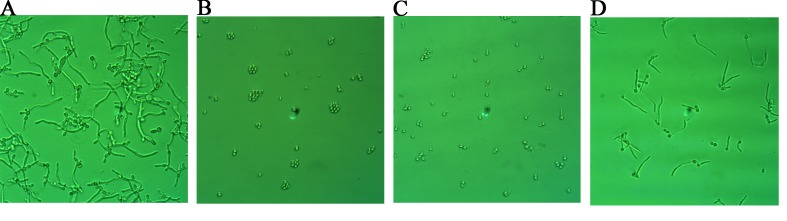
Morphological changes of *C. albicans* after suloctidil treatment **(A)** Control; morphological changes of *C. albicans* at different culture times (0 hour **(B)**, 2 hrs **(C)** and 4 hrs **(D))** exposed to suloctidil (16 μ g/ml). *C. albicans* was cultured for 12 hrs.

In the control group (Figure 4A), after 12 hours of growth, *Candida albicans* hyphae were clearly visible. If suloctidil (16 μg/mL) was added at the beginning of the *C. albicans* culture, its hypha was completely suppressed (Figure 4B); if antibiotics were added after 2 hours of *Candida albicans* culture, only a very small amount of hyphae were observed (Figure 4C). However, suloctidil (16 μg/mL) could not decrease hypha growth after four hours of *C. albicans* culture (Figure 4D).

### Relative expression of *C. albicans* hyphal specific genes

**Figure 5:**
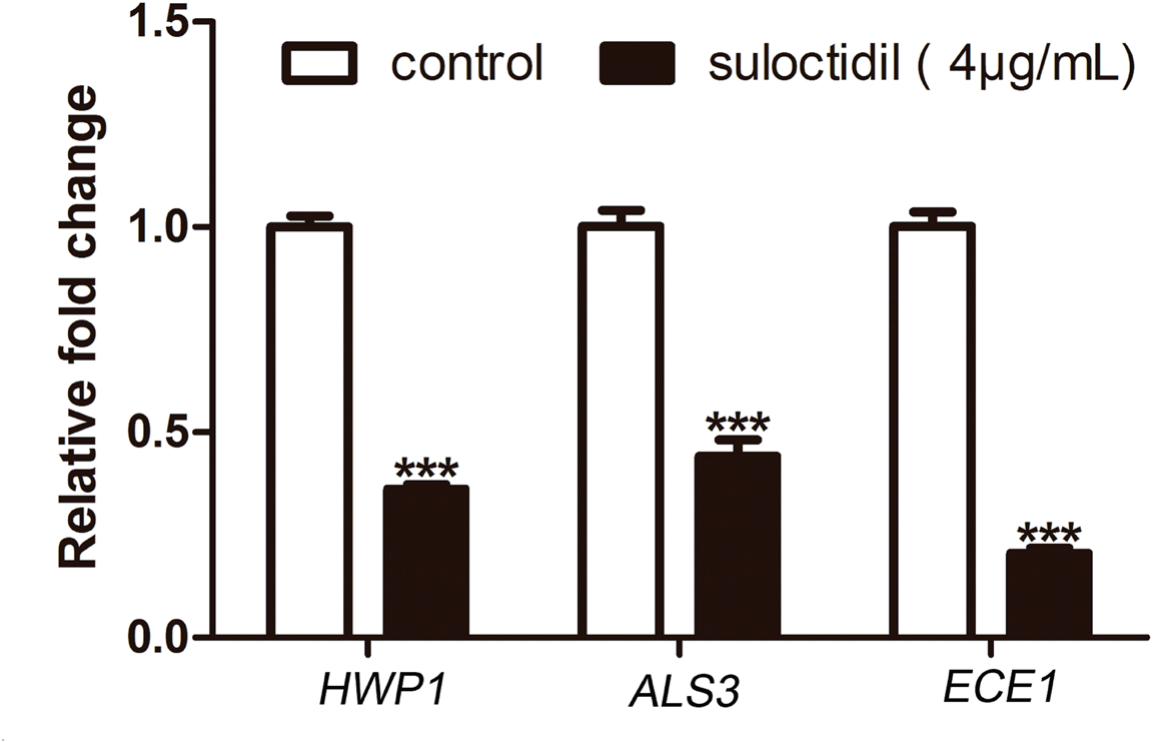
qRT-PCR analysis of expression of hypha-specific genes (*HWP1*, *ALS3*, and *ECE1*) in *C. albicans* treated with suloctidil (4 μg/mL) for 6 hours *P< 0.05; **P < 0.01; ***P < 0.001.

The expression levels of hypha-specific genes, Hyphal Wall Protein 1 (*HWP1*) [6], Agglutinin Like Sequence 3 (*ALS3*) [24], and extent of cell elongation 1 (*ECE1*) [25], in suloctidil-treated cells were significantly reduced by 2.8-fold, 2.3-fold, and 4.9-fold, respectively, compared with those in the control (Figure 5). This result also revealed that suloctidil down-regulated certain hypha-specific genes (*HWP1, ALS3*, and *ECE1*) of *C. albicans*.

### *In vivo* therapeutic efficacy of suloctidil for *C. albicans* infections

**Figure 6:**
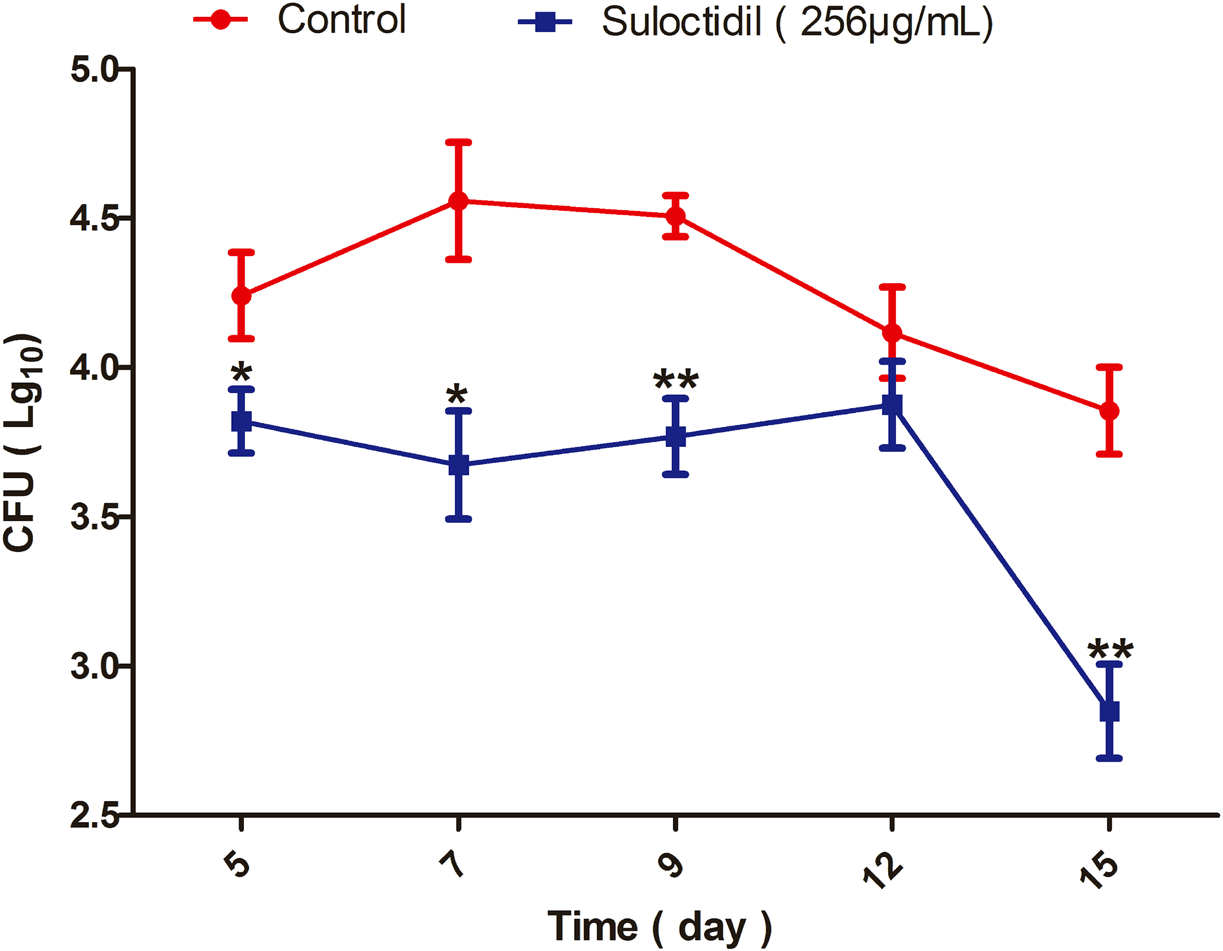
Measurement of *C. albicans* load in mice treated with suloctidil Vaginal lumen of mice under pseudoestrus condition were infected with *C. albicans* YEM30 and treated with suloctidil (256 μg/mL) every day. At 5, 7, 9, 12 and 15 days post-infection, the fungal burden of vaginal lavage fluids was collected and determined via the colony forming units (CFU) assay. A significant difference was observed between the suloctidil-treated group and the vehicle-treated control group *(P* < 0.05). Data are the mean ± SD (n = five mice) for one representative experiment in three independent experiments. *P < 0.05, **P < 0.01.

The *in vivo* antifungal efficacy of suloctidil was further investigated in a murine model of vaginal candidiasis. The results in Figure 6 show a remarkable reduction in the *C. albicans* load of mice treated with suloctidil compared with that of control (PBS)-treated mice, beginning at 5 days post-infection (*P*<0.05). This beneficial effect was maintained until 15 days post-infection, especially at the 9th (*P*<0.01) and 15th days (*P*<0.01).

### Suloctidil-treated mice show reduced inflammatory pathologic changes

**Figure 7:**
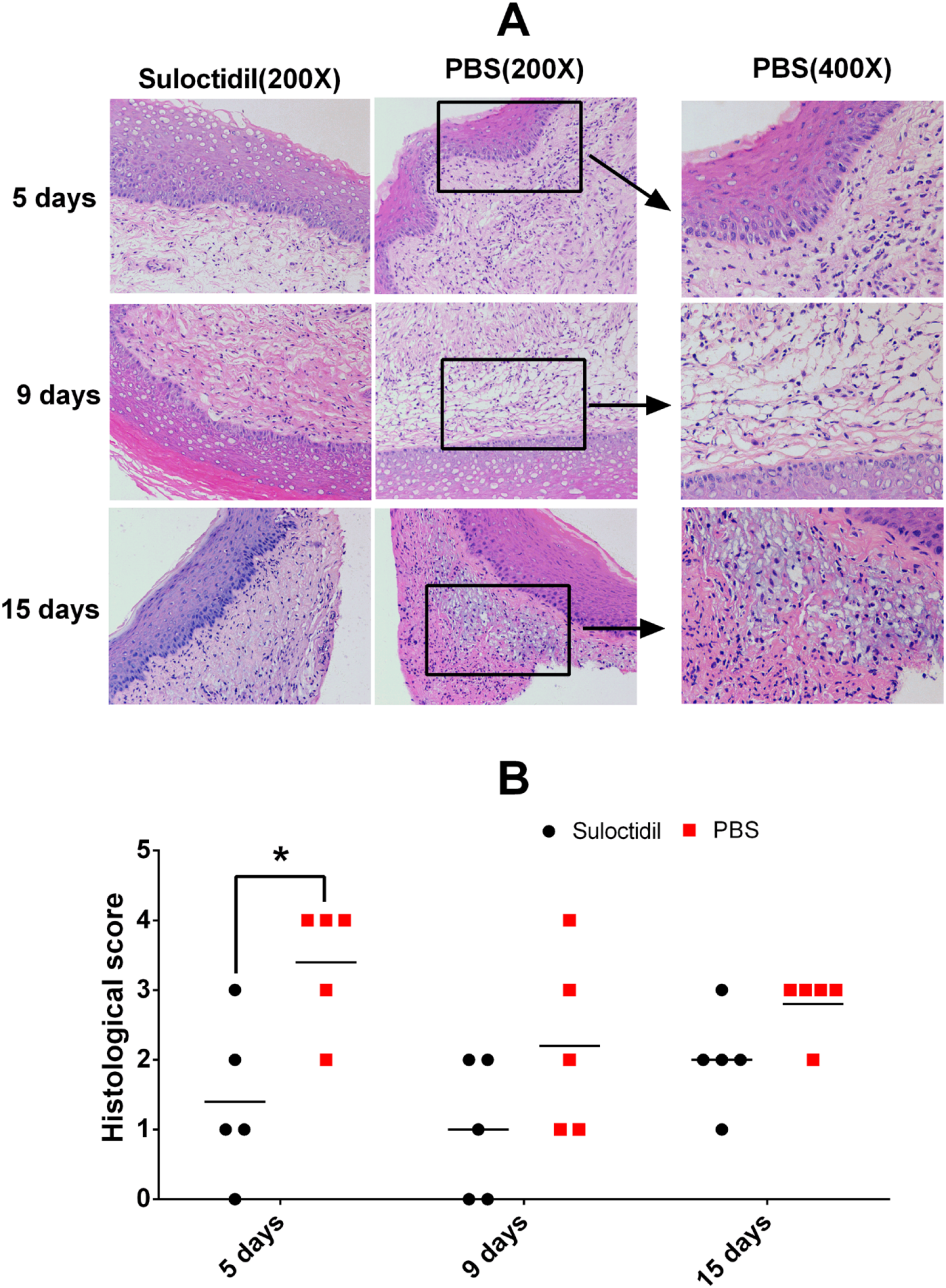
Suloctidil-treated mice show reduced inflammatory pathological changes Vaginal tissues of mice pretreated with suloctidil or vehicle were collected after infection and analyzed for histological changes by hematoxylin and eosin staining under light microscopy. Representative images (Magnification: 200x, 400x) are shown in **(A)**, and areas of lymphocytic inflammation in each section are marked by rectangles. Histopathology scores for vaginal tissues from Kunming mice are shown in **(B)** (n = 5/group, 7–8 weeks old). *P < 0.05.

We compared the histopathological changes in the vaginal tissue between mice with or without suloctidil treatment after infection. At day 5 post-infection, large amounts of cellular infiltration comprising primarily neutrophils and mononuclear cells were found in the mucosa propria and submucosa in the vehicle-treated infected mice; the suloctidil-treated mice showed undetected inflammatory cell infiltration (Figure 7; *P*<0.05). At day 9 post-infection, vehicle-treated infected mice showed significant cellular infiltration, comprising primarily lymphocyte and mononuclear cells in the mucosa propria, and the suloctidil-treated mice showed mild histologic changes (Figure 7). Both groups showed inflammation at day 15 post-infection; however, the suloctidil-treated mice showed less lymphocyte and cellular infiltration in the mucosa propria (Figure 7).

## DISCUSSION

Biofilm formation is a crucial virulence factor of *C. albicans* that is closely associated with its pathogenesis [10, 26]. The development of *C. albicans* biofilms includes three phases: the adhesion phase (0–1.5 h), the initial colonization phase (1.5–25.5 h), and the maturation phase (25.5–19.5 h) [8]. *C. albicans* biofilms formed under clinical conditions have intrinsic resistance to antifungal agents [26].

Previous studies have shown that suloctidil is a calcium-channel blocker [27]. The current study found that suloctidil has antifungal activity and synergy with FLU against *C. neoformans* [14]. However, the inhibitory activity of suloctidil against *C. albicans* biofilms has not been studied. In the present study, the anti-biofilm activities of suloctidil *in vitro* and *in vivo* were examined for the first time. The XTT assay results and SEM images showed significant reduction in metabolic activities and morphological changes of *C. albicans* after suloctidil treatment.

Based on the inhibitory testing results, when 16 μg/mL of suloctidil was administered at the initial phase, almost all *C. albicans* stayed in the yeast phase without transforming to the hyphal phase, which suggested that suloctidil inhibited hyphal growth. Hyphae are a characteristic feature and constitute the structure of biofilm. Thus, hyphal formation and adherence are vital and essential for biofilm development and sustainability [28]. Furthermore, the morphological transition from yeast to hyphae is strongly associated with fungal infection, and the change from the yeast phase to filamentous phase is critical in the pathogenesis of fungi [29]. A previous study showed that *C. albicans* without hyphae-forming ability is non-virulent in mouse models and displays weaker pathogenicity [30]. Thus, hyphal formation and growth, as well as the yeast-hyphae transition phase, are highly relevant to *C. albicans* biofilm formation and pathogenicity. Therefore, suloctidil has an important effect on the treatment of *C. albicans* infection.

The expression profile of hyphal growth-associated genes (*HWP1, ALS3*, and *ECE1*) was analyzed to gain insights into the molecular mechanism of suloctidil-mediated hyphal growth inhibition. Expression profile analysis revealed that suloctidil (4 μg/mL) down-regulated the expression of hyphal genes, which inhibited hyphal growth. *HWP1* is a well-characterized hypha-specific gene that mediates cell-cell interactions and improves the biofilm formation of *C. albicans* [31, 32]. Moreover, *HWP1* regulates the adhesion of *C. albicans* to epithelial cells via the Hwp1 protein [6]. *ECE1* is a hypha-induced gene that is related to cell elongation [31, 33]. *ALS3* is a member of the agglutinin-like sequence (*ALS*) gene family that encodes cell-wall glycoproteins [24]. *ALS3* contributes to cell adhesion, whereas both *ALS* and *HWP1* genes are highly expressed in hyphae and play essential roles in the yeast-to-hypha morphological transition of *C. albicans* [24, 34–37]. The suloctidil-mediated down-regulation of hypha-related genes may be a cause of mycelial growth inhibition, perhaps sufficient even to prevent the biofilm formation of *C. albicans.*

In conclusion, the effect of suloctidil against *C. albicans* biofilm was evaluated *in vitro* and *in vivo*. A low dose of suloctidil (4 μg/mL) inhibited the yeast-to-hypha morphological transition of *C. albicans* as well as the expression of *HWP1, ALS3*, and *ECE1* genes. Suloctidil suppressed the formation of *C. albicans* biofilms by down-regulating the expression of *HWP1, ALS3*, and *ECE1* to prevent the morphological transition from yeast to hypha. Therefore, our results revealed that suloctidil is a potential treatment for superficial biofilm-associated Candidal infections. However, detailed pharmacodynamics and pharmacological studies should be conducted in the future.

## MATERIALS AND METHODS

### Organisms, media, and growth conditions

This study utilized wild type strains of *C. albicans*, YEM30 and LC3, isolated from a patient with vaginal candidiasis from QiLu hospital. All strains were stored in 20% glycerol at -80°C prior to the experiments. The frozen glycerol stock of the strain was regularly revived on YPD agar medium (1% yeast extract, 2% tryptone, 2% glucose, and 2% agar). For the broth culture, strains were grown in YPD medium at 30°C with agitation (100 rpm). RPMI-1640 medium was buffered with morpholinepropanesulfonic acid buffer (Sigma-Aldrich, St. Louis, US) to pH 7. Stock solutions of suloctidil (Sigma-Aldrich, St. Louis, US) were prepared in dimethyl sulfoxide (DMSO, Sigma-Aldrich, St. Louis, US) and stored at -20°C until further use.

### Biofilm development

All strain cells were washed twice with sterile phosphate-buffered saline (PBS) and added to RPMI-1640 medium at a density of 1×10^7^ cell/mL. Biofilm experiments were performed in untreated 96-well cell culture plates. Briefly, 100 μL of cell suspension was added to each well for 1.5 h of adhesion at 37°C with agitation (75 rpm). The wells were washed twice with PBS to remove non-adherent cells. To allow the growth of biofilm, 200 μL of fresh RPMI-1640 medium was added to each well. The plates were incubated in an orbital shaker for 24–48 h at 37°C with agitation (75 rpm). At 24 h of incubation, the medium was aspirated, and the biofilms were washed twice with PBS, followed by the addition of 200 μL of freshly prepared RPMI-1640 medium.

### Suloctidil susceptibility testing

MICs of suloctidil were investigated in 96-well plates with RPMI-1640 medium in accordance with the broth microdilution method for the CLSI standard M27-A2 [15]. Stock solutions of suloctidil at 40 mg/mL were used for MIC studies. Then, 100 μL two-fold dilutions of suloctidil (4-32 μg/mL) were added. Each cell suspension (5×10^6^ CFU/mL) was diluted (1:50 dilution, followed by 1:20 dilution) in RPMI-1640 medium to obtain twice the final inoculum size of 2.5×10^3^ CFU/mL. 100 μL of inoculum was then added, and the plates were incubated for 48 h at 37°C with agitation (75 rpm). MIC_80_ was expressed as the minimal concentration in the well that exhibited 20% growth of planktonic cells compared with that of the positive control wells. Growth was detected as turbidity (492 nm) relative to the uninoculated well using a microtiter plate reader. RPMI-1640 medium was used as a negative control, and overnight-cultured *C. albicans* was used as positive controls in the wells. Selected wells that contained 100 μL of RPMI-1640 with 5% DMSO and without suloctidil were set as the control.

### Effect of suloctidil on *C. albicans* biofilm formation and on pre-formed biofilms

The biofilm formation assay was performed in 96-well plates as previously described, with slight modifications. The cell suspension was prepared in RPMI-1640 medium at a density of 1×10^7^ cells/mL, dispensed into 96-well plates (100 μL per well), and incubated for 1.5 h at 37°C with agitation (75 rpm). After incubation, the cell suspensions were removed, and each well was washed twice with PBS to remove non-adherent cells. Two-fold dilutions of suloctidil in RPMI-1640 medium (100 μL per well) were added to each well. Then, the plates were further incubated for 24 h. Similarly, 100 μL of RPMI-1640 medium that contained 5% DMSO without suloctidil was added into the selected wells for the control.

To obtain preformed biofilms, the medium was aspirated after 48 h of incubation. Non-adherent cells were removed by washing the biofilms twice with sterile PBS. Briefly, 100 μL of two-fold dilutions of suloctidil were added to the wells of prewashed biofilms. The controls were performed as previously described. Furthermore, 96-well plates were incubated at 37°C for 24 h.

The metabolic activity of biofilms was quantitatively determined by colorimetric XTT [2,3-bis-(2-methoxy-4-nitro-5-sulfophenyl)-2H-tetraz- olium-5-carboxanilide sodium salt] reduction assay.

### Oxidative activity assay

Biofilm growth activity was quantified via the colorimetric XTT reduction assay [16, 17]. XTT (Sigma-Aldrich, St. Louis, US) was dissolved in sterile PBS to obtain a 1 mg/mL XTT solution. The solution was sterilized and stored at -70°C until use. Menadione solution (0.4 mM; Sigma-Aldrich, St. Louis, US) was prepared before use. Anhydrous D (+) glucose was dissolved in PBS at a final concentration of 200 mM. The XTT solution was thawed on ice and mixed with the menadione solution at a volume ratio of 20:1. Non-adherent cells were removed by washing the biofilms twice with sterile PBS. Then, 158 μL of glucose, 40 μL of XTT, and 2 μL of menadione were added to each well of the 96-well plates. The plates were covered with aluminum foil and incubated at 37°C for 3 h. Thereafter, 100 μL of the solution was transferred to new 96-well plates. The absorbance was measured at 492 nm using a microtiter plate reader.

### Time-kill curve

The kinetics of the antibiofilm activity of the drugs was investigated using the time-kill test. As previously described, the medium was aspirated at 1.5 h adhesion, followed by the addition of 100 μL of RPMI-1640 medium. The two strains were treated with 100 μL of suloctidil at concentrations of 4, 8, 16, and 32 μg/mL. The 96-well plates were covered with aluminum foil and incubated at 37°C with agitation (75 rpm). At predetermined time points (3, 6, 9, 12, 18, 24 h), the media from the corresponding wells were removed and washed twice with PBS. The metabolic activity of the biofilms was quantitatively determined by colorimetric XTT reduction assay.

Preformed biofilms were developed in 96-well plates as previously described. The medium was aspirated at 48 h of incubation. The biofilms were washed twice with PBS, followed by the addition of 100 μL of fresh medium. Suloctidil was used at concentrations of 32, 64, 128 and 256 μg/mL for both strains. The 96-well plates were covered with aluminum foil and incubated at 37°C with agitation (75 rpm). Each well was aspirated and washed twice with PBS at predetermined time points (0.5, 1, 3, 6 h). The metabolic activity of biofilms was quantified as previously described.

### Microscopic analyses

*C. albicans* biofilms (YEM30) in the mature phase (48 h-old) with or without suloctidil (64 μg/mL) treatment were grown on glass cover slips in 6-well cell culture plates (Corning Inc., New York, US). The glass cover slips were placed in fixative (3% glutaraldehyde in PBS) overnight. After rinsing with PBS and 30 min of immersion in 1% osmium tetroxide, the sample was dehydrated, dried, mounted, sputtered with gold, and visualized under a scanning electron microscope (SEM) (Sigma 300, Carl Zeiss Inc., Oberkochen, Germany).

### Hyphal growth test

Previous studies have reported that hyphal growth is highly associated with *C. albicans* virulence and pathogenesis [18]. We performed the hyphal growth test to verify the antihyphal formation activity of suloctidil by visualizing the morphological changes in the strains. The hyphal growth assay was tested on the hyphal growth of *C. albicans* YEM30. *C. albicans* were developed for 0 h, 2 h and 4 h in different plates. Afterwards, cell suspensions were aspirated, and a total of 1 mL of fresh RPMI-1640 medium and 1 mL of suloctidil (16 μg/mL) was added to the wells. The plates were incubated further for 12 h at 37°C with agitation (75 rpm). Fresh RPMI-1640 medium with DMSO was used as a control. Thereafter, critical images were stored. All images are presented in Figure 4.

### Expression analysis of *C. albicans* hypha-specific genes by qRT-PCR

The effect of sub BIC_80_ of suloctidil on the expression of hypha-specific genes *HWP1, ALS3*, and *ECE1* was evaluated by quantitative real-time reverse transcription PCR (qRT-PCR). *C. albicans* YEM30 suspension was added to RPMI-1640 medium that contained hyphal inducer (5% FBS) at a concentration of 1×10^7^ cell/mL. This test was performed in sterile culture plates (Thermo Fisher, Waltham, USA). First, 1 mL of cell suspension was transferred to culture plates and incubated for 1.5 h at 37°C. After incubation, the cell suspensions were removed, 1 mL of suloctidil (4 μg/mL) was added, and 100 μl of RPMI-1640 medium containing 5% DMSO without suloctidil was used as the control. After 18 h of incubation at 37°C, the plates were washed twice with PBS and transferred to microtubes.

The total RNA of the cells was investigated via the TRIzol method [19]. Following extraction, RNA concentration, purity, and quality were verified using a BioPhotometer plus (Eppendorf AG, Hamburg, Germany). The cDNA was synthesized using a PrimeScript™ RT Reagent Kit with gDNA Eraser (Takara, Kusatsu, Japan) in accordance with the manufacturer’s instructions. Primers for the target (*HWP1, ALS3*, and *ECE1*) and housekeeping internal control (*ACT1*) genes (Table 2) were synthesized by BioCune (Shanghai, China). The cDNA template (100 ng), gene-specific sense and antisense primers (400 nM), and SYBR^®^ Premix Ex Tag (Takara, Kusatsu, Japan) were used in the reaction mixture per the manufacturer’s instructions. The qRT-PCR was performed using a Mastercycler® ep realplex Real-time PCR system. The following parameters were used for qRT-PCR: initial denaturation at 95°C (3 min), followed by 40 cycles of denaturation (95°C/5 s), annealing (60°C/30 s), extension (75°C/45 s), and melting-curve analysis starting from 95°C for 15 s, 60°C for 1 min, and 95°C for 15 s. The specificity of the primers was confirmed using melting curve analysis. The generated C_t_ values of target genes were normalized to the C_t_ value of the housekeeping *ACT1* gene. Relative expression fold changes were evaluated by the ΔΔC_t_ method using a previously published formula [19].

**Table 2:** Primer lists of *ACT1, HWP1, ALS3, ECE1* for qRT-PCR

Primer	Sequence (5’-3’)	Primer	Sequence (5’-3’)
*ACT1*-f	GGTTTGGAAGCTGCTGGTATTGACC	*ACT1*-r	ACGTTCAGCAATACCTGGGAACATG
*HWP1*-f	TGGTGCTATTACTATTCCGG	*HWP1*-r	CAATAATAGCAGCACCGAAG
*ALS3*-f	TACTTCCACAGCTGCTTCCA	*ALS3*-r	GGAGCATTACCACCACCATT
*ECE1*-f	GCTGGTATCATTGCTGATAT	*ECE1*-r	GGAGCATTACCACCACCATT

### Murine vaginal candidiasis model

The murine vaginal candidiasis model was established with Kunming mice following a previously described method with minor modifications [20–22]. Fifty-three female Kunming mice (28–32 g body weight) were used at 7 to 8 weeks of age. The mice were randomly assigned to 2 groups with five mice each: either vehicle or suloctidil and were maintained under a pseudoestrus condition by subcutaneous injection of 0.2 mg of estradiol benzoate (every other day, CEN’S, Hangzhou, China) 7 days prior to infection and until the completion of the study. A *C. albicans* suspension (1×10^7^ cells/mL) in 20 μL of PBS was administered to the vaginas of the mice. The filaments in the mouse vaginas were observed after 7 days of infection. Intravaginal treatment with 50 μL of 256 μg/mL suloctidil was administered into the vaginas of mice every day until day +21. PBS was used as a control. After various treatment times, the mice were sacrificed and the vaginal lumen was thoroughly washed and scraped with 1 mL of PBS. To determine the fungal load in the vagina, 100 μL of lavage fluid from each mouse was plated on YPD agar plus ceftriaxone (50 μg/mL, Roche, Basel, Switzerland). CFUs were then evaluated.

### Histological analysis

The mice vaginas were removed, stored in the same fixative solution (10% buffered formalin), and embedded in paraffin. The vaginal tissue was sliced into 5-μm thick sections for hematoxylin and eosin staining (HE) and examined by light microscopy. Inflammatory cell infiltrates (Polymorphonuclear leukocytes and mononuclear cells) in the antrum and body were graded as described: 0, none; 1, some infiltrates; 2, mild infiltrates (few aggregates in submucosa and mucosa); 3, moderate infiltrates (several aggregates in submucosa and mucosa); 4, marked infiltrates (many large aggregates in submucosa and mucosa); 5, nearly the entire mucosa contained a dense infiltrate; and 6, the entire mucosa contained a dense infiltrate [23].

### Statistical analysis

All assays were performed in triplicate, and each experiment was repeated thrice on independent days unless otherwise stated. A total of 5 repetitions per plate were performed for the murine vaginal candidiasis model experiment. All obtained results were expressed as the mean ± standard deviation. All data sets for the comparison of treated and control groups were analyzed by one-way ANOVA. All analyses were conducted with GraphPad Software (GraphPad Prism® Version 6.0c, La Jolla, CA, USA) at a 95% confidence level.
